# The History and Current Killings of Polio Vaccinators in Pakistan: A Need for Targeted Surveillance Strategy

**DOI:** 10.1177/10105395231158866

**Published:** 2023-03-01

**Authors:** Braira Wahid, Babita Kumari, Khaled Mohammed Saifullah, Muhammad Idrees

**Affiliations:** 1 Department of Microbiology, Monash Biomedicine Discovery Institute, Monash University, Melbourne, Victoria, Australia; 2 Faculty of Health, Engineering and Sciences, University of Southern Queensland, Brisbane, Queensland, Australia; 3 Division of Molecular Virology, Centre of Excellence in Molecular Biology, University of the Punjab, Lahore, Pakistan

**Keywords:** surveillance, polio, endemic, Pakistan, Afghanistan

## Abstract

Poliomyelitis has been eliminated from all countries of the world except Pakistan and Afghanistan. One of the major reasons is the stigmas associated with the polio vaccine that has been repetitively discussed in literature, and governments of both the countries are already making serious efforts to control this public health challenge, but till this moment, the state officials have not introduced any surveillance strategy for the security of polio workers in National Emergency Action Plan (NEAP) for Polio Eradication. This report highlights the issue of targeted killing and terrorism attacks on polio vaccinators in Pakistan and also devises a surveillance strategy to provide security to polio workers at immediate possible because the current chaos in Afghanistan will ultimately lead to more terrorist attacks on polio vaccinators.

## What We Already Know

- Pakistan has shown increase in polio virus cases in the last two years.
- In 2018, 12 cases were confirmed and in 2019 147 cases before declining to 87 in 2020.
- Polio virus strains have been found in sewage water collected from 25 different locations of Karachi.

## What This Article Adds

- The killing of polio workers in Pakistan and Afghanistan is an issue of grave concern.
- There is a lack of awareness of the dangers of polio, and false religious beliefs are quite common in northern areas of Pakistan, especially in cities near the Pakistan-Afghan border.
- More than 200 polio team workers have lost their lives while working on polio campaigns.

Poliomyelitis is a viral infection transmitted via the oral-fecal route and targets the motor nervous system. This can lead to paralysis and is sometimes fatal. The Global Polio Eradication Initiative (GPEI) documented the eradication of polio in 1988 from almost all countries except Afghanistan, Nigeria, and Pakistan. Pakistan has shown increase in polio virus cases in the last two years. In 2018, 12 cases were confirmed and in 2019 147 cases before declining to 87 in 2020. Polio virus strains have been found in sewage water collected from 25 different locations of Karachi. A recent survey conducted by National Emergency Operations Centre (NEOC) also confirmed the presence of polio virus in sewage samples collected from 12 cities of Pakistan, including Rawalpindi, Lahore, Peshawar, Waziristan, Bannu, Sukkur, Kambar, Hyderabad, and Mardan. Pakistan is infected with wild polio virus type I (WPV1), circulating vaccine-derived poliovirus type 1 (cVDPV1), or cVDPV3. The presence of virus anywhere poses serious threats to the health of children and can place significant burdens on the health infrastructure and economy of the country. Several social factors have made the eradication of polio virus a major challenge.

The killing of polio workers in Pakistan and Afghanistan is an issue of grave concern. There is a lack of awareness of the dangers of polio, and false religious beliefs are quite common in northern areas of Pakistan, especially in cities near the Pakistan-Afghan border. A majority of the population is not familiar with the consequences and transmission dynamics of polio virus.^1^ Another population study from Peshawar, Pakistan, reported that 79% of participants were not willing to vaccinate their children as they believe that vaccine was composed of ingredients that are prohibited in their religion.^2^

Pakistan is still endemic for polio because of a long history of attacks on polio workers. The increasing terrorist attacks on polio vaccinators have made its elimination increasingly difficult. During the last decade, security personnel and police accompanying polio workers have also become victims of terrorism attacks. Some local resources have confirmed 70 deaths of polio workers in Khyber Pakhtunkhwa (KPK) province, since 2012.

The year 2021 started with a further shooting incident on polio teams and with parents refusing to vaccinate their children in Karak City of KPK province of Pakistan. Karak is a hotspot of poliovirus because the cases reported from there in 2020. This hostile attitude toward polio vaccinators and misconceptions about religion and polio vaccine may turn out to be a major public health challenge of the century because the rest of the world had been declared polio free and Pakistan may become a source of its transmission to other parts of the world. Although Pakistan has outlined effective strategies under the National Emergency Action Plan to address current challenges, unfortunately, this plan does not include any policy and strategic priority for the safety of polio workers.

**Figure 1.**
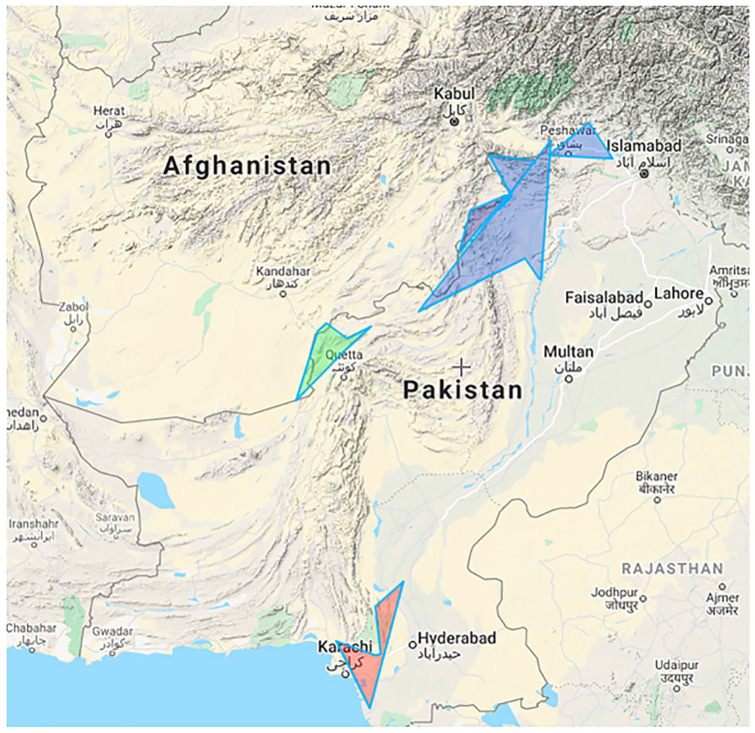
Potential hotspots of terrorist attacks on polio teams (blue highlight indicates high-risk area, red indicates intermediate risk, and red indicates low-risk area).

According to published reports, most of the attacks on polio workers were committed by The Taliban and the 2021 crisis in Afghanistan and the Taliban’s take over will probably lead to more targeted killings of polio vaccine teams. Therefore, it is very necessary for Pakistan to establish and implement an effective surveillance strategy that must address polio eradication along with the safety of polio workers. More than 200 polio team workers have lost their lives while working on polio campaigns, including female workers, male workers, police, and security personnel and a large number of casualties have also been reported. In January 2016, 16 polio workers died in a suicide attack in Quetta while 6 female polio workers were shot and died in the same city. A further source reported 68 deaths all across Pakistan from December 2012 to January 2014. In addition to this, multiple cases of verbal and physical abuse have also been reported in Karachi and KPK province and 11 teachers involved in polio campaigns were also abducted from Khyber agency (Figure 1).

In Table 1, we collected data related to polio workers’ killings from different media sources and observed the highest number of killings in different cities of KPK province, specifically the towns located near the Pakistan-Afghan border. Karachi and Quetta are also potential hotspots of terrorism attacks on polio workers. We also noticed that most of the victims of terrorist attacks were females, reflecting the composition of the polio teams. These statistics and figures may not represent the actual count because large number of cases go unreported due to unavailability of media sources and correspondents in remote and underprivileged parts of the country. Maximum number of killings occurred during 2012 to 2016, and the Taliban claimed responsibility for most of the attacks, and current political chaos in Afghanistan indicates terrorism and insecurity will be a persistent challenge. The absence of any proper strategic plan of security of polio worker in “National Emergency Action Plan (NEAP) for Polio Eradication 2020” will lead Pakistan to a failing trajectory.

**Table 1.** Killings of Polio Workers Confirmed by Local Media Sources.

Date	Number of vaccinators killed	Location
July 2012	Foreign doctor of UN injured Local doctor killed	Karachi
December 18, 2012	1 male killed, 1 injured	Karachi
December 18, 2012	2 females and 2 males killed	Karachi
December 18, 2012	1 male killed	Peshawar
January 31, 2013	2 females killed	Kurram agency
February 28, 2013	18 workers killed 1 policeman killed	Across the country
June 16, 2013	2 polio volunteers were killed	Swabi
October 7, 2013	2 people were killed and over a dozen—including two polio workers—were injured in a bomb blast	Peshawar
October 10, 2013	3 police officers	Peshawar
November 23, 2013	11 teachers carrying out polio vaccinations were kidnapped	Khyber Agency
November 30, 2013	A police official was killed and another injured when unidentified men fired at them	Peshawar
December 13, 2013	1 police officer 2 police guard	Swabi
December 18, 2013	Motorcyclist opened fire on polio team 80 ppl but no one injured	Karachi
December 21, 2013	1 person killed	Khyber agency
December 21, 2013	1 polio supervisor killed	Jamrud
December 28, 2013	2 injured 1 killed	Peshawar
January 21, 2014	2 females killed, 2 escaped attack 1 polio worker killed Car snatched from polio team	Karachi Mansehra Pangore
January 21, 2014	3 males	Karachi
March 1, 2014	12 killed	Peshawar
September 15, 2014	1 male	Pishin
December 9, 2014	1 killed	Faisalabad
January 26, 2015	1 policeman	Karachi
February 5, 2015	Two members of a polio team were injured	Bahadurpur village near Thull town in Jacobabad district
February 17, 2015	Polio worker team guards kidnapped and killed on February 16, two suicide bombers blew themselves up in the same area while briefly encountering security forces during a search operation to find the polio team	Zhob
February 18, 2015	4	Zhob
February 22, 2015	Female worker threatened	Karachi
March 17, 2015	2 women polio workers	Mansehra
March 18, 2015	1 male	Bajaur
September 15, 2015	2 females beaten	Lahore
November 30, 2015	1 male	Swabi
December 22, 2015	1 female gang raped	Peshawar Nowshehra
January 13, 2016	14	Quetta
January 15, 2016	Polio team beaten	Faisalabad
April 21, 2016	7 police officer killed	Karachi
June 28, 2016	Team harassed	Shahidan, Karak
April 19, 2017	2 females were beaten up	Lahore
November 3, 2017	Police arrested two women for allegedly thrashing and manhandling a polio	Multan
January 18, 2018	2 females	Quetta
January 19, 2018	Mother and daughter died Two security personnels	Bannu
January 20, 2018	1 female rape attempt	Muzaffargarh
February 2, 2018	2 females	Quetta
February 18, 2018	1 female abduction	Peshawar
March 19, 2018	1 FC personnel killed 3 abducted workers recovered	Mohmand Agency
November 14, 2018	1 female injured	Swabi
December 26, 2018	1 female injured	Quetta
April 9, 2019	1 Police	Mohmand
April 23, 2019	1 police officer	Quetta
April 23, 2019	Policeman killed Policeman attacked with sharp weapon	Lahore Peshawar
April 24, 2019	1 person shot dead	Buner
April 25, 2019	1 female killed	Chaman
May 6, 2019	1 male dead	Bajaur
June 5, 2019	Crushed by train	Peshawar
January 29, 2020	2 women killed	Swabi
June 9, 2021	1 cop killed	Mardan
August 2, 2021	1 cop killed	DI Khan

**Figure 2.**
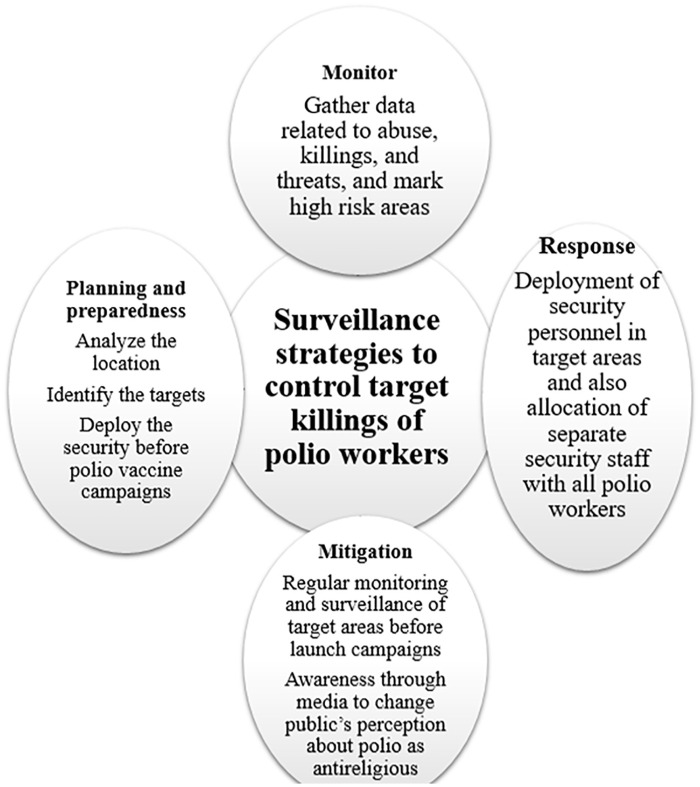
Target surveillance strategy to be included in National Emergency Action Plan (NEAP) for Polio Eradication.

This study highlights the gap in National Emergency Action Plan (NEAP) for Polio Eradication 2020 and also proposes surveillance strategy to control the risk of terrorism attacks on polio teams that are highly probable to occur in the near future following Taliban’s takeover in Afghanistan during the second half of the year 2021 (Figure 2).
